# Diagnostic error increases mortality and length of hospital stay in patients presenting through the emergency room

**DOI:** 10.1186/s13049-019-0629-z

**Published:** 2019-05-08

**Authors:** Wolf E. Hautz, Juliane E. Kämmer, Stefanie C. Hautz, Thomas C. Sauter, Laura Zwaan, Aristomenis K. Exadaktylos, Tanja Birrenbach, Volker Maier, Martin Müller, Stefan K. Schauber

**Affiliations:** 1 Department of Emergency Medicine, Inselspital University Hospital, University of Bern, Freiburgstrasse, 3010 Berne, Switzerland; 2 Max Planck Institute for Human Development, Center for Adaptive Rationality (ARC), Lentzeallee 94, 14195 Berlin, Germany; 3 AG Progress Test Medizin, Charité Medical School, Hannoversche Straße 19, 10115 Berlin, Germany; 4 Institute of Medical Education Research Rotterdam, Erasmus Medical Center, Rotterdam, The Netherlands; 5 Department of General Internal Medicine, Inselspital University Hospital, University of Berne, Freiburgstrasse, 3010 Berne, Switzerland; 6 Centre for Educational Measurement, University of Oslo, Gaustadallén 30, 0373 Oslo, Norway; 7 Centre for Health Sciences Education, Faculty of Medicine, University of Oslo, Oslo, Norway; 8 Skills Lab Lernzentrum, Charité Universitätsmedizin Berlin, Chariteplatz 1, 10117 Berlin, Germany

## Abstract

**Background:**

Diagnostic errors occur frequently, especially in the emergency room. Estimates about the consequences of diagnostic error vary widely and little is known about the factors predicting error. Our objectives thus was to determine the rate of discrepancy between diagnoses at hospital admission and discharge in patients presenting through the emergency room, the discrepancies’ consequences, and factors predicting them.

**Methods:**

Prospective observational clinical study combined with a survey in a University-affiliated tertiary care hospital. Patients’ hospital discharge diagnosis was compared with the diagnosis at hospital admittance through the emergency room and classified as similar or discrepant according to a predefined scheme by two independent expert raters. Generalized linear mixed-effects models were used to estimate the effect of diagnostic discrepancy on mortality and length of hospital stay and to determine whether characteristics of patients, diagnosing physicians, and context predicted diagnostic discrepancy.

**Results:**

755 consecutive patients (322 [42.7%] female; mean age 65.14 years) were included.

The discharge diagnosis differed substantially from the admittance diagnosis in 12.3% of cases. Diagnostic discrepancy was associated with a longer hospital stay (mean 10.29 vs. 6.90 days; Cohen’s *d* 0.47; 95% confidence interval 0.26 to 0.70; *P* = 0.002) and increased patient mortality (8 (8.60%) vs. 25(3.78%); OR 2.40; 95% CI 1.05 to 5.5 *P* = 0.038). A factor available at admittance that predicted diagnostic discrepancy was the diagnosing physician’s assessment that the patient presented atypically for the diagnosis assigned (OR 3.04; 95% CI 1.33–6.96; *P* = 0.009).

**Conclusions:**

Diagnostic discrepancies are a relevant healthcare problem in patients admitted through the emergency room because they occur in every ninth patient and are associated with increased in-hospital mortality. Discrepancies are not readily predictable by fixed patient or physician characteristics; attention should focus on context.

**Trial registration:**

https://bmjopen.bmj.com/content/6/5/e011585

**Electronic supplementary material:**

The online version of this article (10.1186/s13049-019-0629-z) contains supplementary material, which is available to authorized users.

## Introduction

Diagnostic errors are frequent, [1–3] have severe medical [4, 5] and economic [6] consequences, and account for a considerable proportion of legal claims against physicians. [7–9] Emergency medicine is particularly prone to diagnostic error because of its high workload and time pressure, factors competing for attention simultaneously, and potentially life-threatening consequences of wrong diagnoses. Additionally, diagnoses in the emergency room are often based on incomplete and unreliable information. [10, 11]

The U.S. National Academy of Medicine (NAM) recently estimated, that most people will experience at least one “meaningful” diagnostic error in their lifetime, sometimes with devastating consequences. [1] Yet estimates of the size of the problem vary strikingly, [1–3, 12] presumably due to different definitions of diagnostic error [13] as well as the variety of methods that have been used to assess it. [14] Specifically, some definitions focus on an incorrect diagnostic label, regardless of the presence of a process error, [5, 15, 16] while others consider diagnostic errors as a missed opportunity in the diagnostic process. [13, 17] Additionally, most common research methods focus on retrospective analysis of error cases and are vulnerable to documentation bias or selection bias. [18] Finally, studies that heavily rely on expert raters to determine whether a diagnostic error occurred, are susceptible to hindsight [19, 20] and outcome bias. [19, 21] One prominent definition of diagnostic error, which we also employ in this study, is based on the discrepancy between the diagnosis under investigation and a more definitive, later diagnosis: Graber defines diagnostic error as a “diagnosis that was unintentionally delayed [ …], wrong [ …], or missed [ …], as judged from the eventual appreciation of more definitive information”. [15]

In addition to different definitions, a large variety of causes of such diagnostic discrepancies have been identified. [1] However, one limitation of most studies is that they have focused exclusively on cases with diagnostic discrepancies. Without the comparison of cases with and without diagnostic discrepancies, it cannot be determined whether and to what extent the causes identified differentiate between cases with and without diagnostic discrepancies. For instance, several cognitive biases are generally assumed to cause diagnostic error. [22–26] Yet most of these findings are based on retrospective analyses of erroneous cases only or vignette studies in which physicians were tricked into falling prey to cognitive bias. [27–32] It thus remains unclear whether the cognitive processes identified are also at work in correctly diagnosed cases, [33] and whether and to what extent they apply in the clinical workplace. [19, 33–35] Because many diagnoses are first made in the emergency room where diagnostic error is rife, [10] it is of particular importance to understand the clinically relevant factors associated with discrepancies and these discrepancies consequences in emergency care.

Consequently, the purpose of this paper was to

1. calculate the rate of discrepancies between diagnosis at hospital admittance and discharge in patients hospitalized through the emergency room.
2. determine the consequences such discrepancies have.
3. identify factors that predict discrepancies.

## Methods

We conducted a prospective observational study combined with surveys at a university-affiliated tertiary care hospital in Switzerland. Around 45,000 patients present to the hospital’s emergency room per year. [36]

Patients were included prospectively and factors known to affect the diagnostic process (i.e., physician, patient, and context factors [37]) were assessed in clinical practice. After patient discharge from the hospital, we determined whether there was a diagnostic discrepancy between the ERs’ admission diagnosis and discharge diagnoses and compared the characteristics and consequences of the cases with and without diagnostic discrepancy.

### Study procedure

All patients of 18 years or older hospitalized from the emergency room (ER) to any internal medicine (IM) ward were included in the study and followed up until hospital discharge or death. Patients were excluded if admitted to IM for palliative care or for social reasons or if they presented with an acute traumatic injury and were admitted to IM for reasons of age, comorbidities, or surgical ward crowding.

### Data collection

We collected data on five occasions.

1. Prior to patient recruitment, we used a customized questionnaire to collect demographic and professional data from all physicians in the ER (e.g., age, gender, work experience, professional background, and current position) to be able to potentially identify factors related to diagnostic discrepancies rooted in physician characteristics stable over many encounters (such as e.g. experience).
2. Throughout the patient recruitment phase, we continuously tracked the total number of concurrent ER patients, as well as the numbers of patients waiting, of patients admitted but not yet transferred, and of patients on critical care, together with the times of presentation and start and end of ER care in an electronic patient chart (E.Care, Turnhout, Belgium). From those data, we calculated the national emergency department overcrowding scale (NEDOCS [38, 39]) for intervals of 15 min, because overcrowding has previously been associated with adverse events in the ER. [40] We further collected the age, gender, triage category and mode of ER admission of all patients presenting to the ER during the recruitment phase to allow for a comparison of patients included into the study with the overall ER population. We also continuously logged noise levels in dB(A) at the physicians’ workplace in the ER with a sound meter (HD600, Extech Instruments, MA) and extracted average and peak noise over intervals of 15 min because workplace noise has previously been associated with medical error. [41]
3. At admission to an IM ward, we recorded the patient’s primary ER diagnosis and presenting complaint to allow for the identification of patient characteristics as source of diagnostic discrepancies (such as presentation with non-specific complaints [42–44]). Last, to allow for the identification of contextual sources of diagnostic discrepancy, [37] we further recorded the date and time of the admittance decision and of admittance to IM and collected from both the diagnosing resident and the attending ER physician in charge independently in a case-questionnaire their confidence in the diagnosis, familiarity with similar patients, perceived level of case difficulty, their fatigue, and workload, and whether or not there was a language barrier with the patient. We further asked both resident and attending physicians independently to rate how typical they deemed the patient’s presentation for the diagnosis assigned, how well they collaborated on the case, and how familiar they were with each other in general. All questions were presented on a one-page customized questionnaire [45, 46] based on established instruments, [47–50] with responses being given on 5-point Likert scales (Additional files 1, 2, 3, 4, 5, and 6).
4. At hospital discharge or death, we recorded date and time as well as the patient’s current diagnoses. For deceased patients, we recorded the last main diagnoses they were treated for at IM (e.g, when a patient died from circulatory failure due to septic shock due to pneumonia, pneumonia was recorded as IMs main diagnosis).
5. To further address the question whether patients with diagnostic discrepancy are simply sicker a priori and thus potentially more complicated to diagnose and with a higher likelihood of adverse outcomes, we calculated all patient's Charlson comorbidity index [51] as well as counted the number of their medications and the number of their groups of medications (i.e. diuretics). Medication groups were defined by the WHOs’ ATC code taxonomy, 2nd level groups. [52]

### Ethical considerations

The ethics committee of the canton Berne registered the study as a quality evaluation study under No. 197/15 and waived the requirement for informed patient consent. The study protocol was previously published. [53]

### Measures

Primary outcome under investigation of this study is diagnostic discrepancy, secondary outcomes are their consequences (length of hospitalization and mortality).

#### Diagnostic discrepancy

Diagnostic discrepancy was defined as a substantial discrepancy between a patient’s primary admittance diagnosis from the ER and the primary hospital discharge diagnosis, following the frequently used definition by Graber and colleagues. [15] We would argue that the hospital discharge diagnosis from internal medicine is more precise than the emergency rooms admittance diagnosis for several reasons: first, the internist colleagues at IM only get to see a preselected patient population. Thus, the variety of symptoms and diseases they are confronted with (and need to distinguish) is much smaller than in the emergency room. Second, internists are highly specialized in diagnosing and treating precisely this population, while emergency physicians are generalists out of necessity. Third, patients remain on IM wards much longer than in any emergency room. Thus, internists have more time to discuss differential diagnoses, order and evaluate additional tests and discuss their considerations. Most importantly, however, the effect of any treatment based on the emergency rooms diagnosis can be observed at IM. Failure of the patient to improve under treatment may prompt any physician to consider another diagnosis. The patient's discharge diagnosis ultimately is the one diagnosis under which the patient improved enough to be discharged. [53]

Three board-certified internists, (two of whom were also board-certified emergency physicians), each with more than 10 years of professional experience, were recruited as expert raters to classify the relationship between a patient’s primary diagnosis at admittance and at discharge according to a predefined and pretested scheme (Additional files 1, 2, 3, 4, 5, and 6). [53] Table 1 provides an overview of the categories in this classification with examples and frequency of occurrence. All patients were randomly assigned to two of the three raters, who classified their diagnoses independently. Raters were not involved in the diagnosis or treatment of patients enrolled in this study and were blinded to all data other than ER and IM diagnoses (especially length of hospital stay and mortality). Interrater agreement was moderate (kappa = 0.54). Discrepancies were resolved in a meeting of all raters by discussion and consensus.

**Table 1:** Scheme to classify a pair of diagnoses from ER (admission) and IM (discharge), extended from [46]

Outcome	Discharge Diagnosis is	Frequency	Explanation	Example
Without diagnostic discrepancy	Identical	436 (57.7%)	The two diagnoses are either verbatim or medically identical.	
	More precise	190 (25.2%)	The IM discharge diagnosis is more precise than the ER diagnosis (e.g., by adding an established, disease-specific score or the result of a test that was not available at the ER).	*ER diagnosis*: atrial Fibrillation *IM diagnosis*: atrial Fibrillation, CHADS_2_-Score 4
	A complication	36 (4.8%)	The primary discharge diagnosis from the IM was not foreseeable at the time of hospital admission at the ER but became the most prominent during hospitalization.	*ER diagnosis*: hospital acquired Pneumonia *IM diagnosis*: 1) acute septic ischemia of both legs 2) Legionella pneumonia
With diagnostic discrepancy	Hierarchically different	25 (3.3%)	The primary ER diagnosis is listed among the IM discharge diagnoses but is not the primary discharge diagnosis.	*ER diagnosis:* 1) Recurrent falls 2) Gastroenteritis *IM diagnosis:* 1) Femoral neck fracture* 2) Recurrent falls 3) Gastroenteritis
	Diagnostically different	68 (9.0%)	The primary ER diagnosis is not among the IM discharge diagnoses.	*ER diagnosis:* acute on chronic obstipation *IM diagnosis*: acute pancreatitis

*No further falls after admittance. ER: emergency room; IM: internal medicine

#### Presenting complaint, diagnosis and diagnostic group

Because the specificity of the chief complaint at emergency presentation is known to affect diagnostic difficulty and patient outcome, [42, 43] two independent expert raters classified all patients’ presenting complaints as either specific or unspecific, according to a predefined list of specific complaints (Additional files 1, 2, 3, 4, 5, and 6). [42] They further classified the patient’s primary diagnoses at admittance and at discharge according to the International Classification of Diseases (ICD), version 10, to ensure the ICD coding is unaffected by hospital wide coding schemes which may be optimized for revenue instead of accurate reflection of the patient’s condition. [54] A subsample of 100 randomly selected patients were independently classified by both raters to assess the interrater agreement (kappa = 0.96 for complaint; kappa = 0.957 for ICD code). ICD codes were grouped through the clinical classification system (CCS) of the Agency for Healthcare Research and Quality. [55]

### Statistical analyses

Statistical analysis was conducted with R software for statistical computing (Version 3.4.3) and IBM SPSS (Version 21).

#### Sample size

Based on a power analysis with alpha = 0.05, power = 85%, 8 independent predictor variables for the outcome (diagnostic discrepancy yes/no), R = 0.2, and a 15% dropout rate, the necessary sample size was estimated to be 500. Given an average admittance of 2 patients from ER to IM per day, we estimated that the recruitment phase, which began on August 15th 2015, would last 9 months.

#### Missing data, data conversion, and descriptive statistics

For 250 patients, no physician-filled case-questionnaires were available and noise recordings were missing for 272 patients due to a technical failure of the recording device. In the latter case of technical problems, we assumed data to be Missing Completely At Random (MCAR) [56, 57]. This means that we assume that the missingness of specific observations for noise recordings was neither associated to an observed or unobserved variable relevant to the outcome measure. Furthermore, we assumed missing questionnaires to be either missing due to a “slip”, that is, physicians simply forgetting to fill them out, organizational issues (not enough printed forms), and so forth. In these cases, the respective observations would hold the assumption of being missing completely at random. On the other hand, it is plausible to assume that at busier timeslots physicians were not able to fill out the forms due to increased workload. In this case, missingness would be related to observed variables (NEDOCS; noise levels), and data would hold the assumption of being Missing At Random (MAR). In both cases, missingness can be adequately handled by missing data techniques. As a robustness check, we compared estimates obtained by three different methods for handling missing data (maximal likelihood estimation within the mixed effects models, multiple imputation by chained equations (MICE) and imputation by random forests). We used the procedures provided by the R packages MICE [58] and missForest [59] to impute missing data.

The mean noise level and mean emergency department crowding score (NEDOCS) were calculated for each patient individually based on all recordings obtained during that patient’s presence in the ER.

Data are described by mean and standard deviation or frequency or median and interquartile range as appropriate.

#### Statistical tests

We used independent sample *t* testing, Chi^2^ testing, and Mann–Whitney *U* testing as appropriate to compare the groups of patients with and without diagnostic error; we calculated Kendall’s *τ*, Cohen’s *d* or odds ratios (OR) as appropriate. The level of statistical significance was set at *P* < .05.

#### Generalized linear mixed effects models

The propensity of a change in diagnosis as a function of the predictor variables was estimated using generalized linear mixed-effects models with a log link and binomial error distribution. [60] Physicians usually diagnosed multiple patients. That is, per physician, there were multiple records for different cases. Hence, we included a random intercept term for physicians in the analysis.

In summary, the aim of our study was two-fold. First, we aimed at describing possible clinical consequences associated with a change in diagnosis. Second, we aimed at providing a model for predicting the occurrence of such a change. In order to investigate the first aim, we modelled the relation between a change in diagnosis and the clinical outcomes (i.e., length of hospital stay and in-hospital mortality). In this case, clinical outcomes were entered into (generalized) linear regression models as dependent variables, while change in diagnosis was the predictor. Then again, in order to address the second aim, change in diagnosis was the dependent variable and we added sets of predictors according to the theoretical framework delineated above.

## Results

**Fig. 1:**
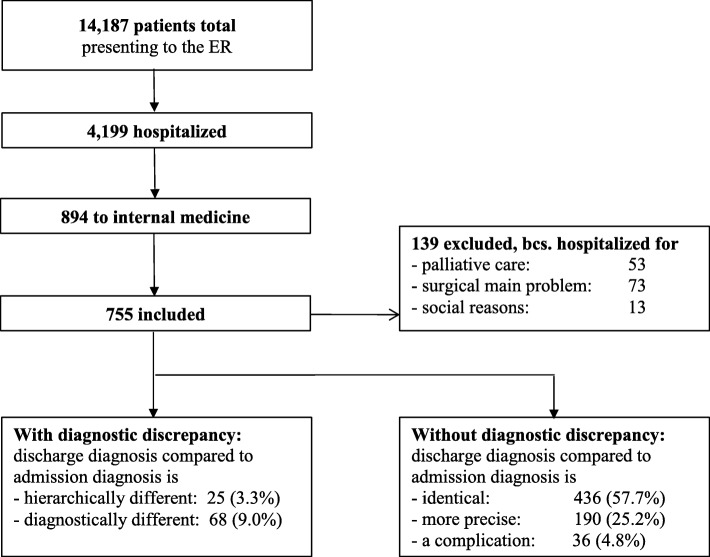
Patient flow and outcome

During the recruitment period, 14,187 patients presented to the ER, of whom 894 were admitted to IM. Of those, 755 were included in the study (Fig. 1). Relative to the general ER population, the groups of hospitalized patients and included patients were older, triaged more urgently, and more often female (Table 2).

**Table 2:** Comparison of general ER population during study period with hospitalized and included patients

	Total	Hospitalized	Hospitalized to IM	Included
Number of patients	14,187	4199	894	755
Female (n [%])	6197 (43.7%)	1684 (40.1%)	425 (47.5%)	322 (42.7%)
Age (years; mean [SD])	48.98 (20.36)	60.63 (18.96)	66.17 (18.08)	65.14 (18.4)
Via resuscitation bay (n [%])	1441 (10.2%)	982 (23.4%)	94 (10.5%)	81 (10.8%)
Triage category (n [%])				
*See immediately*	1068 (7.5%)	740 (17.6%)	43 (4.8%)	43 (5.7%)
*See within 20 min*	3273 (23.1%)	1476 (35.2%)	361 (40.4%)	316 (41.9%)
*See within 120 min*	8386 (59.1%)	1745 (41.6%)	448 (50.1%)	367 (48.6%)
*See today*	924 (6.5%)	152 (3.6%)	29 (3.2%)	23 (3.0%)
*Non-urgent*	540 (3.8%)	86 (2.0%)	13 (1.5%)	6 (0.8%)

All percentages refer to number of patients within column; ER: emergency room; IM: internal medicine

### Relation of change in diagnosis to critical outcomes

A diagnostic discrepancy was identified in 93 (12.3%) of the patients included (Fig. 1 and Table 3). The mortality rate was significantly higher (OR = 2.40; CI = 1.05–5.50) and length of hospital stay (LOS; *d* = 0.47; *p* = 0.002) was significantly longer in patients with a diagnostic discrepancy relative to those without (Table 3). This general pattern also held when we adjusted for possible confounders (i.e., age, sex, Charlson comorbidity index) using (generalized) linear regression models. In this case, mortality rate was still higher (OR_adjusted_ = 2.49; CI = 1.06–5.86) and patients stayed longer in the hospital (standardized Beta_adjusted_ = 0.47; 0.26–0.68). Patients with diagnostic discrepancy were neither older nor sicker (as indicated by triage, Charlson comorbidity index and number of active medications and groups of medication) than those without discrepancy (Table 3).

**Table 3:** Differences between patients with and without diagnostic discrepancy

Measure	Total *n* = 755	Without diagnostic discrepancy *n* = 662 (87.68%)	With diagnostic discrepancy *n* = 93 (12.32%)	*p* ^$^	Effect Size*		
					Type	Estimate	95% CI^§^
At presentation to the ER							
Age (years; mean [SD])	65.14 (18.4)	64.84 (18.68)	67.21 (16.17)	0.199	Cohen’s *d*	0.13	−0.10 – 0.35
Female (n [%])	322 (42.65%)	278 (41.99%)	44 (47.31%)	0.360	Odds Ratio	1.23	0.79–1.89
Non-specific chief complaint (n [%])	165 (21.85%)	143 (21.6%)	22 (23.66%)	0.809	Odds Ratio	0.95	0.62–1.41
Triage category (n [%])							
See immediately	43 (5.7%)	36 (5.44%)	7 (7.53%)	0.281	Kendall’s *τ*	0.04	−0.03 – 0.11
See within 20 min	316 (41.85%)	275 (38.82%)	41 (44.09%)				
See within 120 min	367 (48.61%)	324 (48.94%)	43 (46.24%)				
See today	23 (3.05%)	21 (3.2%)	2 (2.15%)				
Non-urgent	6 (0.8%)	6 (0.91%)	0				
Via resuscitation bay (n [%])	81 (10.73%)	69 (10.42%)	12 (12.9%)	0.463	Odds Ratio	1.28	0.66–2.48
Time at ER (hours; mean [SD])	6.54 (2.97)	6.5 (3.02)	6.87 (2.58)	0.202	Cohen’s *d*	0.13	−0.09 – 0.34
Charlson comorbidity index (points; median [IQR])	4.28 (2.87)	4.27 (2.89)	4.38 (2.76)	0.711	Kendall’s *τ*	0.01	−0.04 – 0.07
Number of active medications (median [IQR])	11.46 (8.94)	11.37 (8.86)	12.04 (9.54)	0.590	Kendall’s *τ*	0.02	−0.05 – 0.08
Number of groups of medications (median [IQR])	3.03 (2.15)	3.02 (2.10)	3.21 (2.40)	0.547	Kendall’s *τ*	0.02	−0.05 – 0.09
Outcome							
Length of hospital stay (LOS) (days; mean [SD])	7.32(7.19)	6.90 (6.56)	10.29 (10.14)	0.002	Cohen’s *d*	0.47	0.26–0.70
Mortality (n [%])	33 (4.37%)	25 (3.78%)	8 (8.60%)	0.038	Odds Ratio	2.40	1.05–5.50

All percentages refer to number of patients within column

^$^ For difference between patients with and without diagnostic discrepancy, two-sided *p* values are reported

* Odds ratio (OR) for dichotomous variables; Kendall’s *τ* for ordinal variables, effect size *d* for metric variables;

^§^ CI is the Confidence Interval, for Kendall’s *τ* this was determined by bootstrapping with 2000 repetitions

### Predicting change in diagnosis by observed variables in the ED

In a first step, we fitted four separate models estimating fixed effects for variables related to (1) patient characteristics, (2) physician characteristics, (3) contexts attributes, and (4) physicians’ evaluations of the diagnostic process to predict diagnostic discrepancy (Table 4). Between-physician variation was negligible in all models (ICC_Physician_ = 0). Physicians’ evaluations of the diagnostic process had a notable effect. Having rated the presentation as atypical predicted later diagnostic discrepancy (OR = 1.95; *P* = .046).

**Table 4:** Results of the generalized linear mixed effect models separate for patient, physician, and contexts attributes, and the diagnostic process

	Patient model			Physician model			Contexts model			Process model		
	OR	CI	*p*	OR	CI	*p*	OR	CI	*p*	OR	CI	*p*
Fixed effects												
*Intercept*	0.05	0.01–0.22	<.001	0.11	0.05–0.29	<.001	0.12	0.08–0.17	<.001	0.21	0.07–0.63	.006
Age	1.22	0.90–1.66	.198									
Gender	1.46	0.83–2.56	.189									
Triage category	1.04	0.69–1.57	.835									
Specific chief complaint	1.32	0.75–2.32	.342									
CCS group*	1.00	0.95–1.07	.868									
Experience				1.06	0.89–1.26	.515						
Gender				1.03	0.59–1.81	.917						
NEDOCS°							0.77	0.53–1.10	.150			
Noise							1.07	0.73–1.55	.734			
Atypical										1.95	1.01–3.74	.046
Confidence										0.80	0.57–1.12	.195
Difficulty										1.04	0.72–1.48	.845
Random effects												
N_Physician_	44			41			43			44		
ICC_Physician_	0.00			0.00			0.00			0.00		

*Diagnostic Group according to Clinical Classification Software [55]; °National emergency department overcrowding scale [38]; LOS = length of hospital stay

In a second step, we successively added the blocks of variables from the first step into one general model that aimed at predicting change in diagnosis based on variables observable in the emergency department (Table 5). Again, physicians rating of typicality was the strongest predictor (OR 3.04; 95% CI 1.33–6.96; *P* = 0.009) and between-physician variation was negligible (ICC = 0). Hence, dropping the random intercept term from the model and fitting a generalized linear model resulted in identical estimates. There was a general tendency that results from using random forest procedures were, largely, comparable to both the multiple imputation or maximum likelihood based approaches. The relation between change in diagnostis and physicians typicality rating was weaker when using random forest based imputation (OR_*missForest*_ = 2.21 vs. OR_*ML*_ = 3.04) and constituted the largest discrepancy across the approaches used. The smallest discrepancy was found for noise levels, which had identical estimates (i.e., OR = 1.05) across the applied techniques. Taken together, physicians rating of typicality was the strongest predictor of diagnostic discrepancy.

**Table 5:** Predicting diagnostic discrepancy by variables obtained in the emergency department

		Model 1		Model 2		Model 3		Model 4		Model 5	
		OR	*p*	OR	*p*	OR	*p*	OR	*p*	OR	*p*
	(Intercept)	0.07	< 0.001	0.05	< 0.001	0.04	0.001	0.05	0.010	0.06	0.056
Patient	Age	1.19	0.252	1.22	0.201	1.21	0.222	1.29	0.183	1.24	0.268
	Gender	1.47	0.179	1.46	0.186	1.48	0.169	1.58	0.214	1.49	0.289
	Triage category			1.05	0.826	1.04	0.858	0.93	0.778	0.95	0.862
	Specific chief complaint			1.31	0.350	1.31	0.353	1.41	0.358	1.62	0.213
	CCS group*			1.00	0.870	1.00	0.870	1.01	0.890	0.98	0.611
Physician	Experience					1.06	0.522	1.15	0.142	1.19	0.092
	Gender					1.01	0.977	0.83	0.627	0.80	0.563
Context	NEDOCS°							0.76	0.126	0.74	0.106
	Noise							1.06	0.778	1.05	0.813
Physicians’ evaluations of the diagnostic process	Atypical									3.04	0.009
	Confidence									0.79	0.345
	Difficulty									1.10	0.697

*Diagnostic Group according to Clinical Classification Software [55]; °National emergency department overcrowding scale [38]

### Detecting diagnostic discrepancy by variables obtained in the emergency department

We investigated the possibility of detecting a diagnostic discrepancy already in the emergency department based on the modelling approach in the previous step. To this aim, we compared the performance of three logistic regression models with respect to their ability to identify cases experiencing diagnostic discrepancy. We fitted a model that only used patient-related variables (age, sex, and triage category) and a model that added physicians rating of the diagnostic process, and the full model form the first step. Those three models were compared to each other with respect to the area under the receiver-operator characteristics curve (AUC) which gives the chance that the model will be able to distinguish between cases in which a diagnostic discrepancy occurs and those in which such a discrepancy will not occur. Importantly, we used a bootstrapping-type approach in this context. Specifically, we split the data randomly into two subsets and used one subset to fit the model, and the other subset to predict occurrence of a discrepancy and to estimate the area under the curve. This routine was repeated 1000 times. On average, AUC for the model including patient-related variables was AUC_patient_ = 0.52. Adding typicality ratings only this rose to, on average, AUC_typicality_ = 0.58. On average, using the full model from the previous step did not further increase the classification accuracy.

## Discussion

In this prospective observational study of patients admitted to an IM ward through the emergency room, we found the primary discharge diagnosis to differ substantially from the ER admittance diagnosis in 12.3% of cases. Patients experiencing such a diagnostic discrepancy were hospitalized for significantly longer and had a significantly higher risk of in-hospital mortality. To the best of our knowledge, this is the first prospective study which links suboptimal diagnoses to patient mortality, although ample previous research has demonstrated the importance of high quality diagnoses for other important outcomes, [15, 17] particularly in emergency care. [8, 9, 11] It however remains an open question whether the diagnostic discrepancies identified in this study are directly causing this increase in mortality or whether patients with more complex diseases, which per se could be at an increased risk of unfavourable outcomes, are also more likely to be misdiagnosed. While patients with and without diagnostic discrepancies did not differ in parameters potentially measuring their complexity (such as triage scale, age, presentation through a resuscitation bay, ED length of stay, Charlson comorbidity index or number of active medications), technically our results demonstrate an association, not a causation.

Our analysis of factors potentially causing diagnostic discrepancy showed that the factors present in cases with such discrepancies differed little from those present in cases without. The percentage of diagnostic discrepancy identified is substantial. Particularly considering the association with LOS and mortality, this study reaffirms the importance of reducing diagnostic discrepancy to improve patient safety. Previous estimates of the frequency of diagnostic error in the ER range from around 12% in a general ER population [18] to 24% or more in selected populations. [61, 62] However, most of these results come from countries that limit the time a patient may be seen in the ER (e.g., to a maximum of 4 h). Patients may then be transferred to a medical investigation unit for a maximum of, say, 24 h and only then admitted to a medical ward. Many European ERs, including the one under investigation here, combine both of these units’ functions within a single ER, [36] thus limiting comparability and likely lowering our estimates of the frequency of diagnostic error relative to ERs operating under a 4-h rule.

The design of this study allows causes and consequences of diagnostic discrepancies to be identified without the influence of hindsight bias or the subjectivity of chart reviewers. Most previous studies used occurrence of a diagnostic error or discrepancy as an inclusion criterion (e.g., [15, 63, 64]), making it impossible to judge to what extent the predictors and consequences of diagnostic discrepancy are also present in correctly diagnosed cases. One Dutch study found inappropriate selectivity in the diagnostic process in 26 of the 34 cases (76%) with diagnostic discrepancy but also in 87 of the 213 cases (41%) without discrepancy. [65] Similarly, the fact that we have not identified significant differences between potential contributing factors in this study may be due to the fact that many of the factors previously associated with error play a role in both, cases with and without diagnostic errors. Further research should try to identify factors that differentiate between cases with and without diagnostic errors. One such factor identified in this study is that emergency physicians seem to sense when their diagnostic reasoning fails: Errors at hospital admittance were predictable by the ER physicians’ judgement that the patient’s presentation was atypical for the primary diagnosis. A previous retrospective record review in internal medicine [15] and a review of diagnostic error in primary care [66] found similar associations. Previous studies also found patient age [67–69], gender [66, 68], or chief complaint [42, 44, 61, 62] to be associated with diagnostic error, others, [67] including ours, did not.

The difficulty in identifying strong predictors of diagnostic error in this study, the limited reproducibility of factors associated with diagnostic error across studies, and our finding that between-physician variation in diagnostic performance is negligible may all be indicative of the importance of context-specificity of diagnostic reasoning. [22, 37, 70] Put briefly, the concept of context-specificity states that performance on a diagnostic task in a given context does not predict performance on a similar task in a different context or on a different occasion. [70, 71] As a consequence, clinicians and clinician-educators need to pay more attention to the circumstances in which errors occur rather than trying to increase a generic general diagnostic ability. [22, 70, 71] According to our findings, simply screening patients (or physicians) for factors predisposing for diagnostic error does not seem a useful approach.

### Limitations

This study investigated discrepancies in diagnoses, not error, which would require a thorough review of the diagnostic process. [13, 16] This limitation at the same time results in the main strength of this study, because as opposed to record reviews, data were collected prospectively, reducing potential documentation and hindsight bias, resulting in a substantially higher interrater agreement on error occurrence than in previous studies. [35, 72] Importantly, this is one of the first prospective studies to compare cases with and without diagnostic discrepancy to assess factors contributing to and consequences of diagnostic discrepancy.

The diagnostic discrepancy rates reported here are probably an underestimation of the true values for several reasons. Specifically, the hospitals’ discharge diagnosis does not necessarily reflect the correct diagnosis. We only followed patients up until hospital discharge, missing diagnostic discrepancy identified after discharge which, previous studies indicate, [62, 64] may be substantial in number. Second, conditions that resolve temporarily through supportive therapy, regardless of whether or not the underlying cause was diagnosed and treated, may have been incorrectly classified as non-discrepant in our study.

Furthermore, the occurrence of missing data is a clear limitation in the current study. Indeed, this is a common issue in many observational studies and statistical approaches have been developed that aim at handling such missingness. However, these methods are themselves based on a number of assumptions that are, in the case of the current study, challenging to investigate empirically. For instance, our analyses are based on the assumption that the missingness in physician-reported questionnaire information is caused either by a mechanism observed in this study or due to random disturbances. While we aimed at providing robust analyses by comparing different methods of handling missing data, none of those methods would account for unobserved confounding variables that might have biased the findings reported here.

Finally, our study only included patients admitted to the IM, a group that was generally triaged as more urgent than the overall ER population. Therefore, our results cannot be generalized to the whole ER population. [68, 73]

### Conclusion

Diagnostic discrepancies occurred in every ninth patient admitted to an IM ward from the ER; it is associated with longer length of hospital stay and higher mortality. Only the diagnosing physicians’ judgement of the patient’s presentation as atypical for the diagnosis predicted error; all other potential predictors were equally present in cases with and without error. Our findings reinforce the importance of context-specificity in diagnostic reasoning. Further studies are needed to identify the rules governing the interaction of patient and physician characteristics with the respective context, in order to identify useful predictors of diagnostic error and develop targeted interventions. [74]

## Additional files

Additional file 1: Physician Case Questionnaire German (PDF 50 kb)

Additional file 2: Classification Scheme for Diagnostic Discrepancies (PDF 170 kb)

Additional file 3: Physician Inclusion Questionnaire English (DOCX 22 kb)

Additional file 4: Physician Case Questionnaire English (DOCX 14 kb)

Additional file 5: List of specific chief complaints (DOCX 15 kb)

Additional file 6: Physician Inclusion Questionnaire german (DOCX 78 kb)
