# Supplementary material for: Diagnostic error increases mortality and length of hospital stay in patients presenting through the emergency room
Source: Scand J Trauma Resusc Emerg Med. 2019 May 8;27:54. doi: 10.1186/s13049-019-0629-z (PMC6505221; doi:10.1186/s13049-019-0629-z)
Supplement: Supplementary file 1 — Physician Case Questionnaire German (PDF 50 kb) [file 13049_2019_629_MOESM1_ESM.pdf]

1 Persönliche Daten

1.1 AKRONYM ARZT (die ersten beiden Buchstaben vom Vornamen der Mutter, die ersten beiden Buchstaben vom Vornamen des Vaters, Tag der eigenen Geburt. Zum Beispiel LIPE05 für Lisa und Peter, eigener Geburtstag am 5. Februar)

1.2 NAME und GEBURTSDATUM des Patienten

2 Die folgenden Fragen beziehen sich auf DIESEN Patienten:

2.1 Wie SICHER sind Sie sich, dass die von Ihnen gestellte Diagnose zutrifft?

unsicher (50%) ☐ ☐ ☐ ☐ ☐ sicher(100%)

2.2 Wie SCHWIERIG war für Sie die Diagnosestellung?

schwierig ☐ ☐ ☐ ☐ ☐ einfach

2.3 Wie VERTRAUT sind Sie mit dem diagnostizierten Krankheitsbild?

nie gesehen ☐ ☐ ☐ ☐ ☐ vertraut

2.4 Wie gross war Ihre ZEITLICHE BEANSPRUCHUNG (durch alle Ihre Patienten) zum Zeitpunkt der Diagnosestellung?

limitierend ☐ ☐ ☐ ☐ ☐ gering

2.5 Wie stufen Sie Ihre MÜDIGKEIT zum Zeitpunkt der Diagnosestellung ein?

sehr müde ☐ ☐ ☐ ☐ ☐ hell wach

2.6 Wie gut konnten Sie sich sprachlich mit dem Patienten VERSTÄNDIGEN?

☐ fliessend ☐ operational ☐ eingeschränkt ☐ bruchstückhaft ☐ über Dritte ☐ gar nicht

2.7 Wie TYPISCH präsentiert sich der Patient im Bezug auf das Krankheitsbild?

☐ atypisch ☐ typisch

2.8 Wie erlebten Sie die ZUSAMMENARBEIT im ärztlichen Team während des diagnostischen Prozesses?

war allein ☐ ☐ ☐ ☐ ☐ sehr gut

2.9 Wie HÄUFIG arbeiten Sie mit den beteiligten ärztlichen Kollegen des UNZ zusammen?

sehr selten ☐ ☐ ☐ ☐ ☐ sehr oft
