# Supplementary figures and images for: Diagnostic error increases mortality and length of hospital stay in patients presenting through the emergency room
Source: Scand J Trauma Resusc Emerg Med. 2019 May 8;27:54. doi: 10.1186/s13049-019-0629-z (PMC6505221; doi:10.1186/s13049-019-0629-z)

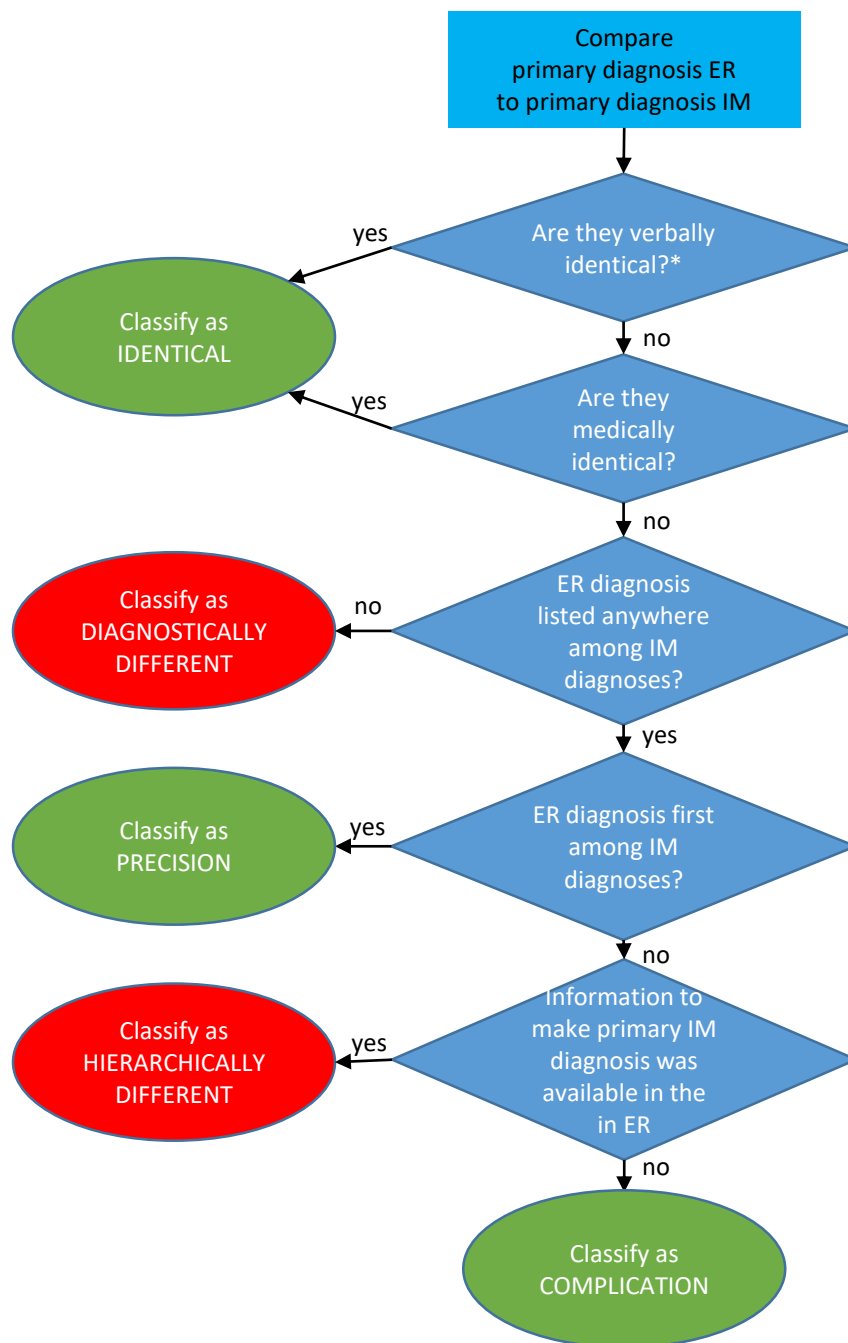

\*Take «suspicion off X», «death because of X» and non-medical specifications of X as X

Supplement: Supplementary file 2 — Classification Scheme for Diagnostic Discrepancies (PDF 170 kb) [file 13049_2019_629_MOESM2_ESM.pdf]
