# Supplementary material for: Diagnostic error increases mortality and length of hospital stay in patients presenting through the emergency room
Source: Scand J Trauma Resusc Emerg Med. 2019 May 8;27:54. doi: 10.1186/s13049-019-0629-z (PMC6505221; doi:10.1186/s13049-019-0629-z)
Supplement: Supplementary file 3 — Physician Inclusion Questionnaire English (DOCX 22 kb) [file 13049_2019_629_MOESM3_ESM.docx]

**Inclusion questionnaire**

**Physicians acronym** ______________________

(the first to letters of your mothers given name, the first two letters of your fathers given name, day of your own birthday. For example, LIPE05 for Lisa and Peter, own birthday on February 5^th^.)

**All the following questions refer to your current educational level / professional standing. Please answer this questionnaire only once. Please return the questionnaire into the studies mailbox in ER section A**

| Your **Gender?** | - Female - Male |
| --- | --- |
| Your **Age?** | _______________________________________ |
| What is your current **function** in the ER? | - Consultant - Resident - Intern |
| Which **specialty training** are you in (or did you already complete)? | - Surgical (surgery, gynecology,…) - Medical (internal medicine, nephrology,…) - General practice - Other - Not decided yet |
| Do you already **have** a **specialty degree**? | - Yes - No |
| How many years of **professional experience** did you already gather since medical school? | ______________________________________ |
| How many years of **professional experience** in **emergency medicine** do you have? | ______________________________________ |

Thank you for your support. With questions or suggestions please contact Wolf Hautz ([wolf.hautz@insel.ch](mailto:wolf.hautz@insel.ch); pager 7879)
