# Supplementary material for: Diagnostic error increases mortality and length of hospital stay in patients presenting through the emergency room
Source: Scand J Trauma Resusc Emerg Med. 2019 May 8;27:54. doi: 10.1186/s13049-019-0629-z (PMC6505221; doi:10.1186/s13049-019-0629-z)
Supplement: Supplementary file 4 — Physician Case Questionnaire English (DOCX 14 kb) [file 13049_2019_629_MOESM4_ESM.docx]

**English translation of the questionnaire filled in by physicians for every patient encounter in the emergency room. Original version in German**

Heading: cDx study – Physician-patient questionnaire

1: Personal Data

- 1. Physicians acronym (the first to letters of your mothers given name, the first two letters of your fathers given name, day of your own birthday. For example LIPE05 for Lisa and Peter, own birthday on February 5^th^.)
  2. Name and date of birth of the patient

2: The following questions refer to THIS patient

2.1 How confident are you, that the diagnosis you assigned is correct? (range from uncertain to certain)

2.2 How difficult was making a diagnosis for you? (range from difficult to easy)

2.3 How used are you to the disease you diagnosed? (range from never seen bevor to used)

2.4 How large was the burden (imposed by all your patients) when you made the diagnosis (range from limiting to little)

2.5 How do you rate your tiredness for when you made your diagnosis? (range from very tired to fully awake)

2.6 How well were you able to communicate with the Patient? (options: fluently, operationally, limited, only pieces, through another person, not at all )

2.7 How typical did the patient present for his disease? (options: atypical, typical)

2.8 How did you experience the collaboration within the healthcare team throughout the diagnostic process? (range from was alone to very good)

2.9 How often do you work with the involved health care professionals at the department of emergency medicine? (range from rarely to regularly)

Bottom line: Thank you for your cooperation. Questions and suggestions may be directed to wolf.hautz@insel.ch, pager 7879
