# Supplementary material for: Diagnostic error increases mortality and length of hospital stay in patients presenting through the emergency room
Source: Scand J Trauma Resusc Emerg Med. 2019 May 8;27:54. doi: 10.1186/s13049-019-0629-z (PMC6505221; doi:10.1186/s13049-019-0629-z)
Supplement: Supplementary file 5 — List of specific chief complaints (DOCX 15 kb) [file 13049_2019_629_MOESM5_ESM.docx]

List of specific chief complaints used in the study. Based on ^1^:

- Pain (chest, abdominal, head, leg, joint, back)
- Dyspnea, cough
- Weakness localized, stroke like symptoms
- Swollen extremity (leg, arm)
- Diarrhea
- Dysuria
- GCS score <14, confusion, intoxication, seizure
- Bleeding (e.g. GI, lung)
- Syncope
- Anxiety, psychotic symptoms, suicidal ideation
- Fever
- Skin lesion, allergic skin reaction
- Vertigo
- Paltpitations
- Nausea with vomiting

The list published by Nemec at al. (2010) further includes trauma as a specific chief complaint. Trauma patients were, however, excluded from the current study.

1. Nemec M, Koller MT, Nickel CH, Maile S, Winterhalder C, Karrer C, et al. Patients presenting to the emergency department with non-specific complaints: the Basel Non-specific Complaints (BANC) study. Acad Emerg Med Off J Soc Acad Emerg Med. 2010 Mar;17(3):284–92.
