# Supplementary material for: Diagnostic error increases mortality and length of hospital stay in patients presenting through the emergency room
Source: Scand J Trauma Resusc Emerg Med. 2019 May 8;27:54. doi: 10.1186/s13049-019-0629-z (PMC6505221; doi:10.1186/s13049-019-0629-z)
Supplement: Supplementary file 6 — Physician Inclusion Questionnaire german (DOCX 78 kb) [file 13049_2019_629_MOESM6_ESM.docx]

**Einschluss-**

**Fragebogen**

*Seite 1 von 1*

**Akronym Arzt** (die ersten zwei Buchstaben vom Vornamen der Mutter, die ersten zwei Buchstaben vom Vornamen

des Vaters, Tag der eigenen Geburt. z.B LIPE05 für Lisa & Peter, eigener Geburtstag am 5. Februar)

# Alle der folgenden Fragen beziehen sich auf Ihren aktuellen Ausbildungsstand / Berufsstand. Dieser Fragebogen ist nur einmalig zu beantworten. Bitte in den Studienbriefkasten Stützpunkt A retour.

Ihr **Geschlecht**? weiblich

männlich

Ihr **Alter**?

Welche **Funktion** haben Sie auf dem UNZ aktuell? OA AA UA

Welche(n) **Facharzttitel** streben Sie an (oder haben Operatives Fach (Chirurgie, Gynäkologie, ...) Sie bereits erworben)? Internistisches Fach (Innere, Nephrologie, ...)

Allgemeinmedizin einen Anderen

noch unentschieden

Sind Sie **bereits Facharzt/Fachärztin**? Ja

Nein

Auf wie viele Jahre ärztlicher **Berufserfahrung** seit

dem Abschlussexamen dürfen Sie zurückblicken?

Auf wie viele Jahre **Berufserfahrung** in der **Notfallmedizin** dürfen Sie bislang zurückblicken (inklusive UNZ)?

Danke für Ihre Mühe. Bei Fragen oder Anregungen zum Fragebogen bzw zur Studie steht Ihnen Wolf Hautz [(wolf.hautz@insel.ch,](mailto:(wolf.hautz@insel.ch) Sucher 7879), stellvertretend für das Studienteam jederzeit zur Verfügung.

Vielen Dank für Ihre Mitarbeit.
